# Validation of MIMGO: a method to identify differentially expressed GO terms in a microarray dataset

**DOI:** 10.1186/1756-0500-5-680

**Published:** 2012-12-12

**Authors:** Yoichi Yamada, Hiroki Sawada, Ken-ichi Hirotani, Masanobu Oshima, Kenji Satou

**Affiliations:** 1 Institute of Science and Engineering, Faculty of Electrical and Computer Engineering, Kanazawa University, Kanazawa 920-1192, Japan; 2 Division of Electrical and Computer Engineering, Graduate School of Natural Science and Technology, Kanazawa University, Kanazawa, 920-1192, Japan; 3 Division of Genetics, Cancer Research Institute, Kanazawa University, Kanazawa, 920-1192, Japan

## Abstract

**Background:**

We previously proposed an algorithm for the identification of GO terms that commonly annotate genes whose expression is upregulated or downregulated in some microarray data compared with in other microarray data. We call these “differentially expressed GO terms” and have named the algorithm “matrix-assisted identification method of differentially expressed GO terms” (MIMGO). MIMGO can also identify microarray data in which genes annotated with a differentially expressed GO term are upregulated or downregulated. However, MIMGO has not yet been validated on a real microarray dataset using all available GO terms.

**Findings:**

We combined Gene Set Enrichment Analysis (GSEA) with MIMGO to identify differentially expressed GO terms in a yeast cell cycle microarray dataset. GSEA followed by MIMGO (GSEA + MIMGO) correctly identified (*p* < 0.05) microarray data in which genes annotated to differentially expressed GO terms are upregulated. We found that GSEA + MIMGO was slightly less effective than, or comparable to, GSEA (Pearson), a method that uses Pearson’s correlation as a metric, at detecting true differentially expressed GO terms. However, unlike other methods including GSEA (Pearson), GSEA + MIMGO can comprehensively identify the microarray data in which genes annotated with a differentially expressed GO term are upregulated or downregulated.

**Conclusions:**

MIMGO is a reliable method to identify differentially expressed GO terms comprehensively.

## Findings

### Background

Microarray technologies allow simultaneous monitoring of the expression of thousands of genes [1]. Many groups have produced microarray datasets for various research topics of interest. Currently, many microarray datasets are deposited in databases such as the Gene Expression Omnibus http://www.ncbi.nlm.nih.gov/geo/[2]. These datasets often contain time-course or tissue microarray data. The first step in the analysis of such microarray datasets often involves the identification of genes whose expression is upregulated or downregulated in specific microarray data when compared with the expression levels in other microarray data [3,4]. Furthermore, to understand the biological implications of differentially expressed genes, biological annotations that are significantly enriched among the differentially expressed genes are often identified. Gene Ontology (GO) and the KEGG PATHWAY database provide over 30,000 biological gene annotations (GO terms) and a few hundred pathway gene annotations, respectively [5,6].

Many tools have been developed to identify the biological annotations that are significantly enriched in differentially expressed genes [7,8]. Of these, Gene Set Enrichment Analysis (GSEA) is a powerful method to determine whether an *a priori*-defined set of genes (e.g., genes annotated with the same GO term) shows statistically significant, concordant expression differences between two distinct microarray data [9].

**Figure 1:**
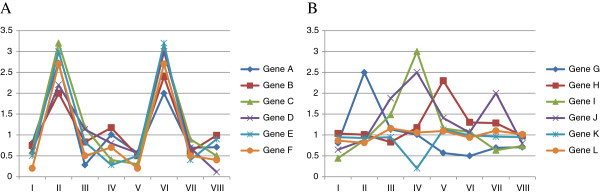
**Differentially expressed GO terms and non-differentially expressed GO terms. A**, an example of a differentially expressed GO term. Identifiers (I–VIII) on the horizontal axis show the microarray data. The vertical axis shows the expression of genes A–F in the microarray data. Genes A–F are annotated with a differentially expressed GO term and are similarly upregulated in microarray data II and VI. Thus, genes that are annotated to a differentially expressed GO term are similarly upregulated or downregulated in specific microarray data when compared with their expression levels in other microarray data. **B**, an example of a non-differentially expressed GO term. Identifiers (I–VIII) on the horizontal axis show the microarray data. The vertical axis shows the expression of genes G–L in the microarray data. Genes G–L are annotated with a non-differentially expressed GO term and are upregulated in mutually different microarray data (i.e., discordant upregulation). Thus, genes that are annotated with a non-differentially expressed GO term are upregulated or downregulated in mutually different microarray data.

**Figure 2:**
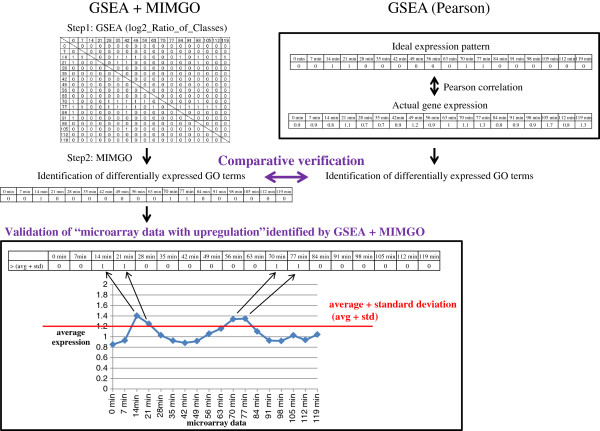
**Validation-flow chart of GSEA + MIMGO.** Eighteen row- and 18 column-indices labeled by time-points (0, 7, 14, etc.) in a matrix of step 1 show the time-course microarray data. GSEA determines whether a gene set assigned a GO term is differentially expressed between row- and column-indices in the matrix of step 1. Log2_Ratio_of_Classes is used as a metric for GSEA. In step 2, MIMGO identifies differentially expressed GO terms using the matrix of step 1. To investigate whether the “microarray data with upregulation” identified by GSEA + MIMGO are correct, the results obtained from GSEA + MIMGO are compared with the average expression level of genes annotated with a GO term for each microarray result. In the square below, the average expression of a gene set annotated with a GO term exceeds the sum of its average and standard deviation only at 14 min, 21 min, 70 min, and 77 min. Therefore, these time-points are marked with 1 in “> (avg + std)”. GSEA (Pearson) shows GSEA using Pearson correlation as metrics. Ideal expression pattern in the time-course microarray data (0 min, 7 min, 14 min, etc.) is prepared as a phenotype label. Pearson correlation coefficients are calculated between the ideal expression pattern and actual gene expressions. The GO term associated with a gene set showing a high Pearson correlation is identified as a differentially expressed GO terms. Finally, “GSEA + MIMGO” is compared with GSEA (Pearson) for identification of true differentially expressed GO terms.

Furthermore, there are several methods (i.e., gene set analysis methods) to identify GO terms that commonly annotate genes whose expression is upregulated or downregulated in particular microarray data compared with their expression levels in other microarray data. Hereafter, we refer to these GO terms as “differentially expressed GO terms” (see Figure 1). As mentioned above, one of these methods is annotation-enrichment analysis after identification of differentially expressed genes, but this has several problems [9-11]. Although the other methods do not have such problems, these others require pre-specification (e.g., 1 for a disease and 0 for a normal) of microarray data in which genes annotated with a differentially expressed GO term are upregulated or downregulated, or cannot identify these microarray data [9-15]. Hereafter, we refer to these microarray data with the differential expression as “de_microarray_data”. To address these issues, we previously proposed an algorithm that can identify not only differentially expressed GO terms but also de_microarray_data [16]. We named this algorithm “matrix-assisted identification method of differentially expressed GO terms” (MIMGO). For each GO term, MIMGO initially prepares a matrix that mutually compares each microarray data pair in a microarray dataset (Figure 2). Then, GO terms that commonly annotate differentially expressed genes between each pair of microarray data are identified using a tool such as GSEA (Figure 2). Finally, using the matrix, MIMGO identifies differentially expressed GO terms and de_microarray_data (Figure 2).

In our previous report, we applied a simple fold change method to identify differentially expressed genes between each microarray data pair in a yeast cell cycle microarray dataset, and tested the statistical significance of GO term annotations to the differentially expressed genes [16]. Ultimately, MIMGO identified differentially expressed GO terms and de_microarray_data. However, we estimated its accuracy only for a few GO terms [16]. Therefore, before MIMGO can be applied reliably to an actual microarray dataset using all available GO terms, it should be validated as to whether it can correctly identify differentially expressed GO terms and de_microarray_data.

Here, we combined GSEA with MIMGO to identify differentially expressed GO terms in a yeast cell cycle microarray dataset.

## Methods

### Microarray dataset

To identify differentially expressed GO terms, we used a time-course microarray dataset in which yeast cells were synchronized by α-factor, as in our previous study [16,17]. Yeast cells were periodically recovered after release from G1 arrest using α-factor. Asynchronous yeast cells growing under the same culture conditions were recovered at the same time-points for use as controls. RNA from experimental and control yeast cells was extracted using the same method. Fluorescently labeled cDNA was synthesized from each extracted RNA, and the ratio of experimental to control cDNA was measured at each recovery time-point. The expression ratio of each gene was subjected to logarithmic conversion. These logarithmic values were returned to the former values by the exponential function with base 2. The dataset includes 6,019 genes and 18 microarray data.

### GSEA

GSEA software was downloaded from the Gene Set Enrichment Analysis website [ http://www.broad.mit.edu/gsea/downloads.jsp]. The GO terms (*N* = 3,474) and their associated yeast genes for GSEA were prepared in a file using the gene_association.sgd and gene_ontology.1_2.obo files downloaded from the Gene Ontology website [ http://www.geneontology.org/]. For the GSEA parameters, “1000”, “gene_set”, “weighted”, and “log2_Ratio_of_Classes” were selected as “Number of permutations”, “Permutation type”, “Enrichment statistic”, and “Metric for ranking genes”, respectively.

GSEA was conducted for each recovery time-point-derived microarray data pair (e.g., 0 min vs. 7 min, 0 min vs. 14 min, 105 min vs. 119 min, 112 min vs. 119 min) from the yeast microarray dataset synchronized by α-factor (see MIMGO below). GO terms (i.e., upregulated GO terms) showing a false discovery rate (FDR) *q*-value below a threshold were identified for each microarray data pair.

### MIMGO

**Figure 3:**
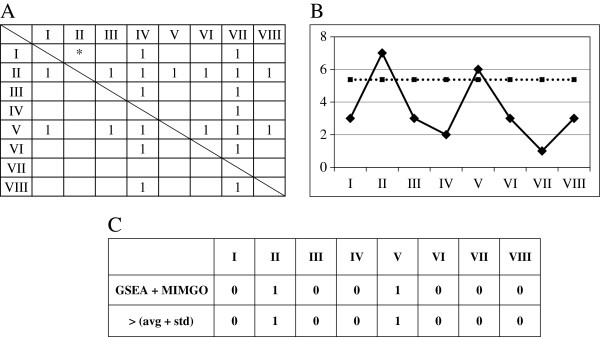
**GSEA + MIMGO and validation of the identified “microarray data with upregulation”. A**, Matrix for pair-wise comparison between microarray data. This matrix is prepared for GO term “**A**”. Identifiers (I–VIII) in the first row and column show the microarray data. The asterisk describes the relative fold inductions of each gene in microarray data I to II. The cells marked with 1 indicate that GO term “**A**” annotates significantly more upregulated genes in its row identifier than in its column identifier. **B**, The average expression of genes annotated with GO term “**A**” in each microarray result. Each number (I–VIII) on the horizontal axis represents a microarray result. The solid line represents the average expression of genes annotated with GO term “**A**” in each microarray result. The dotted line describes the sum of the average and the standard deviation for the average expression (i.e., diamond marks on the solid line) of genes annotated with GO term “**A**” in all the microarray data (I–VIII). C, Validation of “microarray data with upregulation” identified by GSEA + MIMGO. Each number (I–VIII) represents a microarray result. When a statistically significant number of cells marked with 1 is in a row of a matrix as in Figure 3A, the corresponding cell of “GSEA + MIMGO” in Figure 3C is marked with 1. Microarray data that show a value above the dotted line in Figure 3B are marked with 1 in “> (avg + std)” of Figure 3C.

To perform pair-wise comparisons between the microarray data, we prepared a matrix for use in MIMGO for each GO term, as shown in Figure 3A. Suppose that the matrix in Figure 3A is prepared for GO term “A”, and eight microarray data (I–VIII) are compared with each other in the matrix. Following calculation of all gene expression ratios in the row index to the column index for each cell in a matrix, GSEA for the GO term assigned to the matrix was conducted in each cell, excluding those cells marked with a diagonal line. For example, following calculation of all gene expression ratios in microarray data I to II, GSEA for a gene set that is assigned to GO term “A” would be conducted in the asterisked cell of Figure 3A.

When a GO term showed a *q*-value of GSEA below a threshold, the corresponding cell in the matrix prepared for that GO term was marked with 1. For instance, when GO term “A” showed a *q*-value of GSEA below a threshold in microarray I to II in Figure 3A, the asterisked cell in Figure 3A would be marked with 1. Similarly, the same process was repeated in the other cells, except for self-comparisons. In the example, the same process would be repeated in all cells except those marked with a diagonal line in Figure 3A.

To examine whether cells marked with 1 are enriched in any rows compared with in the whole matrix, we used Fisher’s exact test. Here, the null hypothesis is that the proportion of cells marked with 1 out of the total number of cells in a particular row is not different from that in the complement of that row. The null hypothesis was rejected if *p* showed < 0.05 in the following equation:

(1) $$p-\mathrm{value}=\sum_{j=x}^{\min\left(n,M\right)}\frac{{}_{n}C_{j}\cdot_{N-n}C_{M-j}}{{}_{N}C_{M}}$$

in which *N* is the number of cells in the matrix except the self-comparisons, *M* is the number of cells marked with 1 in the *N*, *n* is the number of cells in a row except the self-comparisons, and *x* is the number of cells marked with 1 in the row. An FDR correction was applied to the results of these multiple comparisons using the following equation:

(2) $$\mathrm{False}\;\mathrm{discovery}\;\mathrm{rate}\;\left(\%\right)=\frac{100\times \mathrm{MC}\times p}{\mathrm{PN}}$$

in which *MC* is the number of multiple comparisons, *p* is the *p*-value in equation 1, and *PN* is the number of rows that displayed a *p*-value less than 0.05 in equation 1. Rows showing an FDR lower than 5% were identified as rows significantly enriched with 1. For example, because cells marked with 1 are enriched in the rows of microarrays II and V (Figure 3A), the rows of microarrays II and V show *p*-values of 8.39E-05 and 2.17E-03, and FDRs of 0.0335% and 0.866%, respectively. Thus, GO term “A” is identified as a differentially expressed GO term that is upregulated in microarray data II and V. Similarly, GO terms showing at least one significantly enriched row of cells marked with 1 are identified as differentially expressed GO terms.

Note that there are two multiple comparisons in GSEA + MIMGO: one is multiple comparisons by GSEA, the other is multiple comparisons by Fisher’s exact test in MIMGO. The FDR in equation (2) refers only to the multiple comparisons by Fisher’s exact test in MIMGO.

### Validation of “microarray data with upregulation” and “microarray data with no upregulation” identified by GSEA + MIMGO

To investigate whether “microarray data with upregulation” (de_microarray_data) identified by GSEA + MIMGO were correct, we compared our results with the average expression level of genes annotated with a GO term in each microarray result (Figure 2). For this, the average expression of genes assigned a GO term was first calculated for each time-point-labeled microarray result (e.g., 0 min, 7 min, 14 min). For example, the solid line in Figure 3B shows the average expression of genes annotated with GO term “A” in microarray data I–VIII. Then, the average (Avg_of_A) and standard deviation (Std_of_A) of the average expression levels (diamond marks on the solid line) were calculated. For example, the dotted line in Figure 3B shows the sum of Avg_of_A and Std_of_A in microarray data I–VIII. If the average expression of genes annotated with a GO term was higher than the sum of Avg_of_A and Std_of_A, the corresponding microarray result was marked with 1. For example, because the average expression in microarray data II and V is higher than the sum (dotted line) of Avg_of_A and Std_of_A in Figure 3B, the cells of II and V are marked with 1 in “> (avg + std)” of Figure 3C. On the other hand, a row of cells significantly enriched with 1 in MIMGO was marked with 1 in a comparison matrix such as that shown in Figure 3C. For example, microarray data significantly enriched for cells marked with 1 in Figure 3A (i.e., II and V) are labeled with 1 in “GSEA + MIMGO” of Figure 3C. Then, the Pearson correlation coefficient was calculated between “GSEA + MIMGO” and “> (avg + std)” in Figure 3C. This calculation was conducted for all GO terms identified as differentially expressed GO terms by MIMGO. A high correlation coefficient in this calculation suggests that the de_microarray_data identified by GSEA + MIMGO are roughly valid. Because “> (avg + std)” in Figure 3C does not necessarily correspond to correct answers, this validation of GSEA + MIMGO simply serves as an indication, and does not necessarily connote a sufficient condition.

### Comparison of GSEA + MIMGO and GSEA (Pearson) for the identification of true differentially expressed GO terms

To compare GSEA + MIMGO with GSEA (Pearson), a method that uses Pearson's correlation as a metric, we selected three sets of GO terms as true differentially expressed GO terms from all the GO terms (*N* = 3,474). The first was a set of GO terms (*N* = 7) in which half of their associated genes show r1 ≥0.6 and the other half show r1 <0.6, where r1 is the Pearson correlation coefficient of the gene expression and the vector “0 0 1 1 0 0 0 0 0 0 1 1 0 0 0 0 0 0” (1 only for 14 min, 21 min, 70 min, and 77 min). The second was a set of GO terms (*N* = 22) in which half of their associated genes show r2 ≥0.6, where r2 is the Pearson correlation coefficient of the gene expression and the vector “0 1 1 1 0 0 0 0 0 0 0 0 0 0 0 0 0 0” (1 only for 7 min, 14 min, and 21 min). The third was a set of GO terms (*N* = 5) in which half of their associated genes show r3 ≥0.6, where r3 is the Pearson correlation coefficient of the gene expression and the vector “1 0 0 0 0 0 0 0 0 0 0 0 0 0 0 0 0 0” (1 only for 0 min). When separate GO terms were found to annotate an identical gene set, they were merged into one GO term. Furthermore, when a GO term was found to annotate fewer than three genes, it was removed from the list of true differentially expressed GO terms.

We then examined whether these two methods could detect these true differentially expressed GO terms.

GSEA + MIMGO was conducted for all the GO terms (*N* = 3,474), including the three GO term sets, using a GSEA *q*-value threshold of 0.05. When any row in the matrix showing an FDR lower than 5% in equation (2) was identified for each GO term, we determined that GSEA + MIMGO detected that GO term as a differentially expressed GO term.

GSEA (Pearson) was also conducted for all the GO terms (*N* = 3,474) using three continuous phenotype labels (ideal gene expression): “0 0 1 1 0 0 0 0 0 0 1 1 0 0 0 0 0 0” for GO terms upregulated at 14 min, 21 min, 70 min, and 77 min; “0 1 1 1 0 0 0 0 0 0 0 0 0 0 0 0 0 0” for GO terms upregulated at 7 min, 14 min, and 21 min; and “1 0 0 0 0 0 0 0 0 0 0 0 0 0 0 0 0 0” for GO terms upregulated at 0 min (Figure 2). When each of the three sets of GO terms showed GSEA *q*-values less than 0.05 in the corresponding continuous phenotype label, we determined that GSEA (Pearson) identified those GO terms as differentially expressed GO terms.

## Results and discussion

We used GSEA followed by MIMGO (GSEA + MIMGO) on a yeast cell cycle microarray dataset [17] to identify differentially expressed GO terms. Because the expression level of many genes periodically oscillates during the cell cycle, we expected to identify many differentially expressed GO terms that annotate these genes.

Figure 2 shows a flow chart for GSEA + MIMGO and its validation in comparison with other methods. We first prepared a matrix for each GO term (Figure 3A). Because the yeast cell cycle microarray dataset contains 18 microarray data obtained at 7-min intervals, the matrices contain 18 row- and 18 column-indices labeled by time-point (0 min, 7 min, 14 min, etc.). GSEA was then performed on each row index to each column index of the matrices, and when the GO term assigned to the matrix showed an GSEA *q*-value below the threshold, the corresponding cell was marked with 1 (Figure 3A). Then, MIMGO was applied to each matrix, and GO terms that had at least one row enriched for cells marked with 1 were identified as differentially expressed GO terms (Figure 3A). A row enriched with cells marked with 1 meant that genes associated with the GO term assigned to that matrix showed higher expression in that microarray result than in other microarray data. Conversely, a column enriched with cells marked with 1 denoted that genes associated with the GO term assigned to that matrix showed lower expression in that microarray result than in other microarray data.

“Number of GO terms” in Table 1 shows the number of differentially expressed GO terms identified at each *q*-value threshold of GSEA. As the GSEA *q*-value threshold increased, more differentially expressed GO terms were identified.

**Table 1:** **Validation of all differentially expressed GO terms identified by GSEA + MIMGO at each GSEA *q*-value threshold**

**GSEA q-value threshold**	**0.02**	**0.05**	**0.1**	**0.2**
number of GO terms	302	452	687	1024
average correlation	0.65	0.67	0.67	0.66
standard deviation	0.25	0.23	0.25	0.25
minimum correlation	−0.16	−0.13	−0.15	−0.19
correlation >0.9	53	91	131	194
correlation <0.1	14	14	28	43

“GSEA q-value threshold” shows the *q*-value thresholds used in GSEA. “number of GO terms” denotes the total number of differentially expressed GO terms identified at each GSEA *q*-value threshold. “average correlation” represents the average correlation coefficient between “GSEA + MIMGO” and “> (avg + std)” in Figure 3C for all differentially expressed GO terms identified at each GSEA *q*-value threshold. “standard deviation” describes the standard deviations of the correlation coefficient for all differentially expressed GO terms identified at each GSEA *q*-value threshold. “minimum correlation” shows the minimum of the correlation coefficient for all differentially expressed GO terms identified at each GSEA *q*-value threshold. “correlations >0.9” and “correlations <0.1” show the total numbers of differentially expressed GO terms showing the correlation coefficient over 0.9 and under 0.1, respectively, for all differentially expressed GO terms identified at each GSEA *q*-value threshold.

In contrast to other methods, GSEA + MIMGO can identify microarray data (de_microarray_data) in which genes annotated with a differentially expressed GO term are upregulated. Accordingly, we verified the de_microarray_data using an indicator obtained from another method.

For this reason, we first prepared a matrix equivalent to Figure 3C for each differentially expressed GO term. We then calculated the average and the standard deviation of the average expression levels (diamond marks on the solid line in Figure 3B) for the gene set annotated with a differentially expressed GO term in the 18 microarray data. When the average expression in each microarray result exceeded the sum of their average and standard deviation, the corresponding cells were labeled with 1, as shown by “> (ave + std)” in Figure 3C. On the other hand, rows enriched with cells marked with 1 in the matrix of Figure 3A were also marked with 1, as shown by “GSEA + MIMGO” in Figure 3C. Then, the Pearson correlation coefficient was calculated between “GSEA + MIMGO” and “> (avg + std)” for each differentially expressed GO term (see Figure 3C). Note that we converted the vector of average gene expression across the microarray data to a bit vector of “upregulation” (i.e., 1) and “no upregulation” (i.e., 0) because “microarray data showing upregulation”, but not the expression difference between microarray data showing upregulation or no upregulation, is important to validate the de_microarray_data from GSEA + MIMGO. Furthermore, because “> (avg + std)” generally reminded us of significant upregulation in part of a microarray data set, we used the sum of the average and standard deviation as an indicator of upregulation. The resulting high correlation coefficient suggests that GSEA + MIMGO could identify upregulation of differentially expressed GO terms at roughly correct microarray data.

“Average correlation” in Table 1 showed similar high correlations (0.65–0.67) at all *q*-value thresholds of GSEA. In contrast, as shown in “correlations >0.9” and “correlations <0.1” in Table 1, the total numbers of correlation coefficients above 0.9 and lower than 0.1 tended to increase upon an increasing *q*-value threshold of GSEA. Because a GSEA *q*-value threshold of 0.05 showed better values for “average correlation”, “standard deviation”, “minimum correlation”, and “correlations <0.1” than the other GSEA *q*-value thresholds did, it may be relatively superior at identifying differentially expressed GO terms (Table 1). Therefore, when we compared GSEA + MIMGO with GSEA (Pearson), we used a GSEA *q*-value threshold of 0.05 for GSEA + MIMGO (see Figure 2).

Although it is difficult to determine the enrichment of cells marked with 1 in each row of a matrix in Figure 3A at each GSEA *q*-value threshold, the results from “GSEA + MIMGO” and “> (avg + std)” showed the average correlation higher than 0.65 (*p* < 0.05) at all GSEA *q*-value thresholds (Table 1). This result suggested that GSEA + MIMGO could correctly identify de_microarray_data.

All the matrices of Figure 3C for differentially expressed GO terms identified at GSEA *q*-value thresholds 0.02 and 0.2 are described in Additional file 1 (Additional file 1: Table S1).

Because the total number of differentially expressed GO terms identified varied considerably with the GSEA *q*-value threshold (Table 1), we examined whether the differentially expressed GO terms identified at a GSEA *q*-value threshold of 0.02 were also identified at other *q*-value thresholds (Table 2). Although some differentially expressed GO terms showed no statistical significance in any matrix row at some *q*-value thresholds, at least 286 GO terms showed an enrichment of cells marked with 1 in any matrix row at each GSEA *q*-value threshold (see “number of GO terms” in Table 2). In contrast to Table 1, “average correlation” in Table 2 increased upon an increasing *q*-value threshold of GSEA. Thus, Tables 1 and 2 suggest that the best *q*-value threshold varies by the differentially expressed GO term.

**Table 2:** **Validation of differentially expressed GO terms identified at a GSEA *q*-value threshold of 0.02**

**GSEA q-value threshold**	**0.02**	**0.05**	**0.1**	**0.2**
number of GO terms	302	289	288	286
average correlation	0.65	0.72	.074	0.76
standard deviation	0.25	0.22	0.22	0.21
minimum correlation	−0.16	−0.11	−0.13	−0.19
correlation >0.9	53	72	77	82
correlation <0.1	14	5	4	3

We examined whether the 302 differentially expressed GO terms (0.02_de_GO_terms) identified at a GSEA *q*-value threshold of 0.02 were also identified at the other GSEA *q*-value thresholds. “GSEA q-value threshold” shows the *q*-value thresholds used in GSEA. “number of GO terms” denotes the total number of 0.02_de_GO_terms identified at each GSEA *q*-value threshold. “average correlation” represents the average correlation coefficient between “GSEA + MIMGO” and “> (avg + std)” in Figure 3C for all 0.02_de_GO_terms identified at each GSEA *q*-value threshold. “standard deviation” describes the standard deviation of the correlation coefficient for all 0.02_de_GO_terms identified at each GSEA *q*-value threshold. “minimum correlation” shows the minimum of the correlation coefficient for all 0.02_de_GO_terms identified at each GSEA *q*-value threshold. “correlation >0.9” and “correlation <0.1” show the total numbers of 0.02_de_GO_terms showing the correlation coefficient over 0.9 and under 0.1, respectively, for all 0.02_de_GO_terms identified at each GSEA *q*-value threshold.

**Figure 4:**
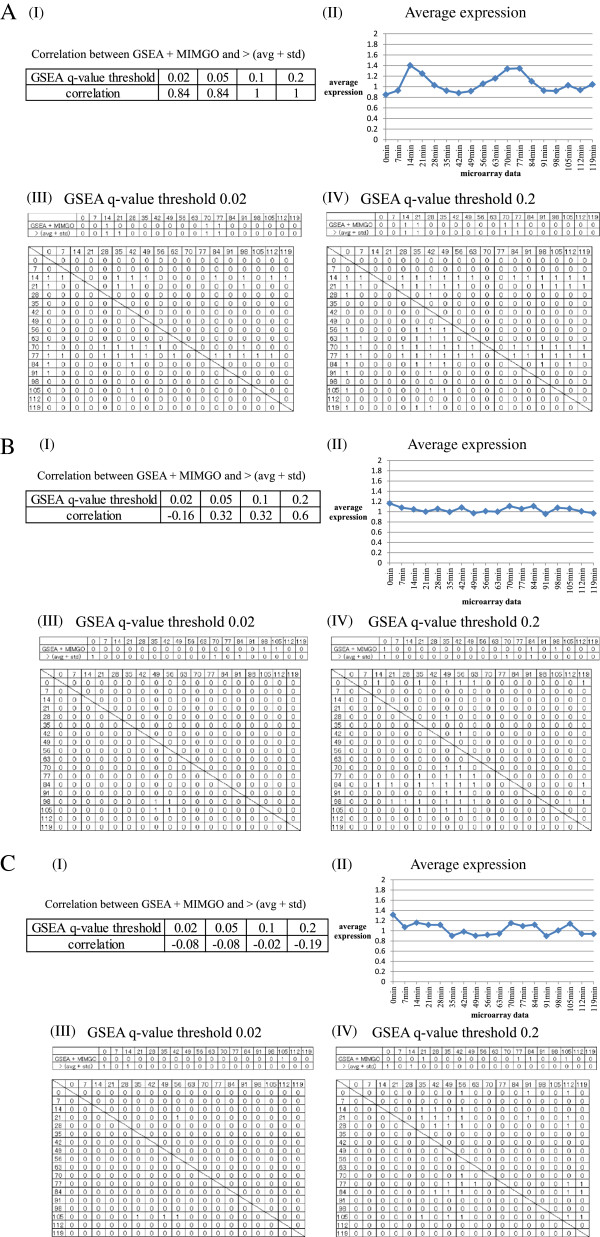
**Detailed validation of results from GSEA + MIMGO for three GO terms.** Detailed results from GSEA + MIMGO for three GO terms (**A**, S phase; **B**, Cellular cell wall organization; **C**, Regulation of cell shape) are shown. Each (**I**) in A–C shows the correlation coefficient between “GSEA + MIMGO” and “> (avg + std)” in Figure 3C for the three GO terms at each GSEA *q*-value threshold. Each (**II**) in A–C describes the average expression of genes annotated with the three GO terms in each microarray result (i.e., 0–119 min). The upper and lower diagrams in each (**III**) describe the matrices of Figure 3C and 3A, respectively, for the three GO terms at a GSEA *q*-value threshold of 0.02. The upper and lower diagrams in each (**IV**) depict the matrices of Figures 3C and 3A, respectively, for the three GO terms at a GSEA *q*-value threshold of 0.2. The 0, 7, 14, etc. in (**III**) and (**IV**) denote the recovery time-point labels for each microarray result.

An example of differentially expressed GO terms in which the average correlation increases upon an increasing *q*-value threshold of GSEA is shown in Figure 4A. Figure 4A shows detailed validation of results from GSEA + MIMGO for the GO term “S phase” that annotates 20 genes. Because “S phase” displays two relatively distinct peaks in the average expression of its associated genes across the time-course (Figure 4A-(II)), 14 min, 21 min, 70 min, and 77 min in “> (avg + std)” of Figure 4A-(III) and -(IV) are marked with 1. A GSEA *q*-value threshold of 0.02 is too stringent for “S phase” to have enough cells marked with 1 in the “21 min” row in the bottom matrix of Figure 4A-(III). Accordingly, the cell marked with 21 in “GSEA + MIMGO” of Figure 4A-(III) has a value of 0. In contrast, a GSEA *q*-value threshold of 0.2 is minimally stringent so that “S phase” showed enough cells marked with 1 in the “21 min” row in the bottom matrix of Figure 4A-(IV), resulting in “1” in the “21 min” cell of “GSEA + MIMGO” of Figure 4A-(IV). Furthermore, when the expression of genes annotated with “S phase” was examined separately, it displayed two concerted upregulations across a microarray dataset, similar to that in Figure 1A.

Thus, the higher average correlation at higher *q*-value thresholds in Table 2 may result from fewer cells marked with 1 in the matrices equivalent to Figure 3A for a subset of differentially expressed GO terms at lower *q*-value thresholds. The alternative view is that this result may be simply because of the increased number of cells containing “1” in a “GSEA + MIMGO” row. Because the vector for “> (avg + std)” generally has few “1” values, the number of GO terms with a correlation coefficient below zero tends to decrease when the vector “GSEA + MIMGO” contains many “1” values.

Finally, we examined two differentially expressed GO terms that displayed minimum correlations at GSEA *q*-value thresholds of 0.02 and 0.2 in Table 1. The GO term that displayed the minimum correlation of −0.16 at a GSEA *q*-value threshold of 0.02 in Table 1 was “Cellular cell wall organization”, which annotates 213 genes (Figure 4B). “Cellular cell wall organization” showed a high correlation of 0.6 only at a GSEA *q*-value threshold of 0.2 (Figure 4B-(I)) and a mild expression change across the time-course (Figure 4B-(II)). Although a few cells are marked with 1 in the bottom matrix of Figure 4B-(III), “GSEA + MIMGO” showed “1” in completely different cells from “> (avg + std)” in the upper matrix of Figure 4B-(III), unlike Figure 4A-(III). The algorithm difference between “GSEA + MIMGO” and “> (avg + std)” could cause this difference, but it is suggested that GO terms that have few cells marked with 1 in the matrices equivalent to Figure 3A should not be identified as differentially expressed GO terms because they display upregulation for only a portion of the microarray dataset (Figure 4B-(III)). In this case, only the set with a stricter threshold in equation (2) cannot prevent MIMGO from identifying “Cellular cell wall organization” as a differentially expressed GO term because there are only a few cells marked with 1 in the bottom matrix of Figure 4B-(III). For example, when there are six cells marked with 1 in the bottom matrix of Figure 4B-(III) and three cells marked with 1 in a row, the *p*-value for the row in equation (1) is 0.0025. Furthermore, when each of the remaining three cells marked with 1 is in a different row, the FDR for the row in equation (2) is 0.38%. We cannot generally set such a strict threshold for the FDR. Therefore, MIMGO should be performed only when a matrix such as that in Figure 3A has a sufficient number of cells marked with 1.

In contrast, the total number of cells marked with 1 increases in the bottom matrix of Figure 4B-(IV) compared with that of Figure 4B-(III), resulting in markings of 1 at roughly equal time-points between “GSEA + MIMGO” and “> (avg + std)” in the upper matrix of Figure 4B-(IV). This also shows that a *q*-value threshold of 0.02 is too stringent for “Cellular cell wall organization” to show “1” in appropriate cells in the upper matrix of Figure 4B-(III). However, when the expression of genes annotated with “Cellular cell wall organization” was examined separately, none displayed any concerted upregulation across the microarray dataset (data not shown). In this case, because there are many cells marked with 1 in the bottom matrix of Figure 4B-(IV), the set that uses a stricter threshold in equation (2) prevents MIMGO from identifying “Cellular cell wall organization” as a differentially expressed GO term at a GSEA *q*-value threshold of 0.2.

The GO term that displayed the minimum correlation (−0.19 at a *q*-value threshold of 0.2 in Table 1) was “Regulation of cell shape”, which annotates 17 genes (Figure 4C). Furthermore, the correlation coefficient at all GSEA *q*-value thresholds showed negative values (Figure 4C-(I)). When the expression of genes annotated with “Regulation of cell shape” was examined separately, none displayed any concerted upregulation across the microarray dataset, similar to in Figure 1B. As previously explained, to avoid identifying “Regulation of cell shape” as a differentially expressed GO term at a lower GSEA *q*-value threshold, MIMGO should be performed only when a matrix such as that in Figure 3A has a sufficient number of cells marked with 1; for “Regulation of cell shape” there are only a few cells marked with 1 in the bottom matrix of Figure 4C-(III). In addition, the set that uses a stricter threshold in equation (2) will prevent MIMGO from identifying “Regulation of cell shape” as a differentially expressed GO term at a higher GSEA *q*-value threshold because there are relatively many cells marked with 1 in the bottom matrix of Figure 4C-(IV).

We next examined whether GSEA + MIMGO can identify true differentially expressed GO terms. Furthermore, we compared its results with those generated using GSEA (Pearson) that used Pearson's correlation as a metric, as shown in Figure 2. As shown in Table 3, the proportions of true differentially expressed GO terms identified were low for both methods, indicating the general disadvantage of GSEA for detecting this type of true differentially expressed GO terms. Dinu et al. also indicated that GSEA is poor at detecting differentially expressed GO terms that equally annotated both highly correlated genes and weakly correlated genes with an ideal expression pattern [13]. Accordingly, the low power of both methods may result from the GSEA algorithm.

**Table 3:** Comparison of GSEA + MIMGO and GSEA (Pearson) for the identification of three sets of true differentially expressed GO terms

	**7 GO terms upregulated at 14min, 21min, 70min, 77min**	**22 GO terms upregulated at 7min, 14min, 21min**	**5 GO terms upregulated at 0min**
GSEA + MIMGO	2 / 7 (28%)	7 / 22 (31%)	1 / 5 (20%)
GSEA (Pearson)	2 / 7 (28%)	11 / 22 (50%)	1 / 5 (20%)
GO terms identified in both methods	1	6	1

The sets of GO terms are described in detail in the text. “GSEA + MIMGO” and “GSEA (Pearson)” denote proportion of true differentially expressed GO terms that were detected by GSEA + MIMGO and GSEA (Pearson) for each set of true differentially expressed GO terms, respectively. ”GO terms identified in both methods" shows the number of the true differentially expressed GO terms identified in both GSEA + MIMGO and GSEA (Pearson).

Both methods were able to identify a similar proportion of true differentially expressed GO terms for each set of GO terms prepared (Table 3). However, GSEA (Pearson) was superior to GSEA + MIMGO in its identification rate of true differentially expressed GO terms for the second set ("22 GO terms upregulated at 7 min, 14 min, and 21 min"). In addition, almost all the true differentially expressed GO terms identified were identical between the two methods (Table 3). This suggests that GSEA + MIMGO was comparable to, but in some instances slightly less powerful than, GSEA (Pearson) at identifying differentially expressed GO terms (Table 3). However, GSEA + MIMGO can comprehensively identify differentially expressed GO terms without the need to set continuous phenotype labels such as “ideal expression pattern” (Figure 2). On this point, GSEA + MIMGO has an advantage over GSEA (Pearson).

**Figure 5:**
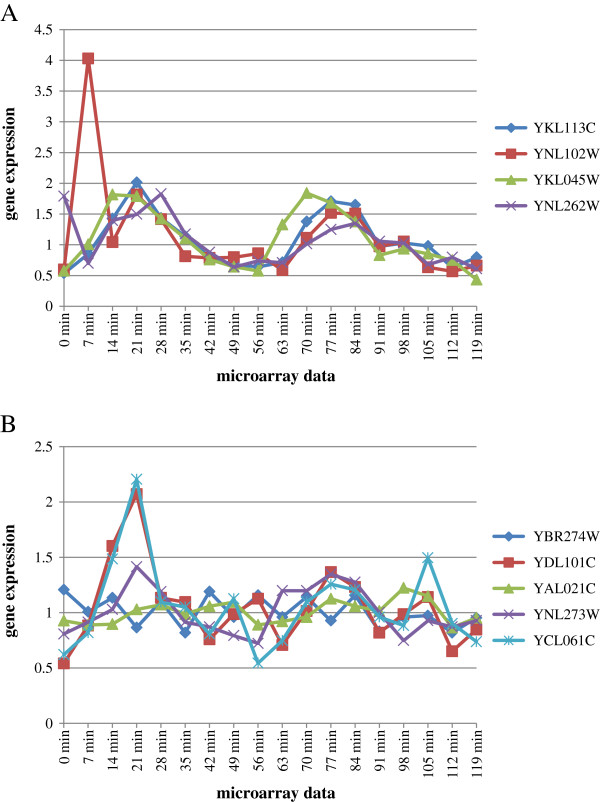
**Two true differentially expressed GO terms identified by either GSEA + MIMGO or GSEA (Pearson). A**, expression of genes annotated to “DNA synthesis involved in DNA repair”. This is a true differentially expressed GO term that GSEA + MIMGO, but not GSEA (Pearson), was able to identify in the first set (“7 GO terms upregulated at 14 min, 21 min, 70 min, and 77 min”) of Table 3. The vertical axis shows the expression of genes (YKL113C, YNL102W, YKL045W, and YNL262W) annotated with “DNA synthesis involved in DNA repair” in the microarray data. **B**, expression of genes annotated with “Replication fork protection”. “Replication fork protection” is a true differentially expressed GO term that GSEA (Pearson), but not GSEA + MIMGO, was able to identify in the first set (“7 GO terms upregulated at 14 min, 21 min, 70 min, and 77 min”) of Table 3. The vertical axis shows the expression of genes (YBR274W, YDL101C, YAL021C, YNL273W, and YCL061C) annotated with “Replication fork protection” in the microarray data.

Figure 5 shows two true differentially expressed GO terms (A: “DNA synthesis involved in DNA repair”, B: “replication fork protection”) identified by either GSEA + MIMGO or GSEA (Pearson) for the first set (“7 GO terms upregulated at 14 min, 21 min, 70 min, and 77 min”) in Table 3. GSEA + MIMGO, but not GSEA (Pearson), was able to identify “DNA synthesis involved in DNA repair” as a differentially expressed GO term (Figure 5A). The expression of YKL113C and YKL045W in Figure 5A showed a high correlation of 0.70 and 0.80, respectively, with the vector “0 0 1 1 0 0 0 0 0 0 1 1 0 0 0 0 0 0”. Conversely, YNL102W and YNL262W in Figure 5A showed a low correlation of 0.14 and 0.32, respectively, with the vector “0 0 1 1 0 0 0 0 0 0 1 1 0 0 0 0 0 0”. Thus, GSEA (Pearson) could not often identify true differentially expressed GO terms that equally annotated genes with high correlation and genes with low correlation with an ideal expression pattern (see Additional file 2: Table S2 ). Consequently, GSEA (Pearson) showed a high GSEA *q*-value of 0.23 for “DNA synthesis involved in DNA repair” (Figure 5A). However, genes annotated to “DNA synthesis involved in DNA repair” showed clearly concerted expression across time-course microarray data (Figure 5A). Unlike GSEA (Pearson), the quantitative expression difference of genes annotated to a GO term between a microarray result and other microarray data is measured in “GSEA + MIMGO”. Therefore, GSEA + MIMGO identified only 21 min and 28 min, which show the most concerted upregulation, to be “microarray data with upregulation”.

On the other hand, GSEA (Pearson), but not GSEA + MIMGO, was able to identify “Replication fork protection” as a differentially expressed GO term (Figure 5B). Genes annotated to “Replication fork protection” did not show any clearly concerted upregulation across the time-course microarray data compared with Figure 5A (Figure 5B). For example, YDL101C and YCL061C, but not YBR274W, YAL021C, and YNL273W, are significantly upregulated at 14 min compared with in other microarray data (Figure 5B). This might explain why GSEA + MIMGO did not identify “Replication fork protection” as a differentially expressed GO term.

In contrast, YDL101C (r = 0.69), YCL061C (r = 0.63), and YNL273W (r = 0.62), which show high correlation with the ideal expression pattern (0 0 1 1 0 0 0 0 0 0 1 1 0 0 0 0 0 0), might result in a relatively low *q*-value of 0.0099 in GSEA(Pearson) for “Replication fork protection” (Figure 5B). Although YNL273W did not show any remarkable upregulation across the microarray data, unlike YDL101C and YCL061C, its expression pattern across the microarray data was similar to the ideal expression pattern. Thus, concerted and large expression differences in genes between microarray data are not necessary important for GSEA (Pearson), unlike for GSEA + MIMGO. These differences between GSEA + MIMGO and GSEA (Pearson) may contribute to the slightly superior power of GSEA (Pearson) in the detection of true differentially expressed GO terms.

All the true differentially expressed GO terms used in this study and the expression of their associated genes in the yeast cell cycle microarray data are described in Additional file 2: Table S2).

In summary, GSEA + MIMGO was able to correctly identify microarray data in which genes annotated to differentially expressed GO terms were upregulated (average correlation ≥ 0.65 (*p* < 0.05)). However, stricter thresholds in equation (2) may have to be applied to GSEA + MIMGO to avoid false-positive identification of differentially expressed GO terms at a higher GSEA *q*-value threshold. In addition, MIMGO should be performed only when a matrix such as that in Figure 3A has a sufficient number of cells marked with 1. Otherwise, the use of a lower *q*-value threshold for GSEA could result in the misidentification of differentially expressed GO terms, because this often leads to few cells marked with 1 in a matrix such as that in Figure 3A.

GSEA + MIMGO is comparable to, or slightly less effective than, GSEA (Pearson) for the identification of true differentially expressed GO terms. However, unlike other methods including GSEA (Pearson), GSEA + MIMGO can comprehensively identify differentially expressed GO terms without pre-specification of the microarray data in which genes annotated with a differentially expressed GO term are upregulated or downregulated. Most often, researchers cannot pre-select microarray data with a phenotype of interest from a microarray dataset before gene set analysis. For example, researchers usually cannot pre-define microarray data in which gene sets are differentially expressed in a time course- or a tissue-microarray dataset before gene set analysis, because they hope to identify gene sets that are differentially expressed in any microarray data of a microarray dataset. In such cases, GSEA + MIMGO is useful for the identification of differentially expressed GO terms, because there is no need to pre-specify the microarray data in which gene sets annotated with GO terms are upregulated or downregulated before gene set analysis. Although the ANOVA, MANOVA, and ANCOVA methods also do not require pre-specification of the microarray data with a phenotype of interest before gene set analysis, they cannot identify microarray data in which gene sets are differentially expressed, unlike GSEA + MIMGO [12,18]. On these points, GSEA + MIMGO has advantages over other gene set analysis methods.

Note that GSEA + MIMGO also has several drawbacks: 1) it does not give any correlation information between differential expression and gene–gene relationships (e.g., pathways) within an *a priori*-defined and differentially expressed gene set, in contrast to pathway analysis tools such as ingenuity pathways analysis [19]; and 2) it assumes the independence of genes within each pre-defined gene set, but this assumption is false for microarray data: it does not account for gene–gene correlation [20]. The resolution of these drawbacks of GSEA + MIMGO is an issue for the future.

## Competing interests

The authors declare that they have no competing interests.

## Authors' contributions

YY, KS, KH, MO, and KS contributed to the conception, design and drafting of the manuscript. YY was responsible for the acquisition, analysis and interpretation of data. All authors have read and approved the final manuscript.

## Supplementary Material

Additional file 1 **Table S1.** The Table shows the comparison matrices of GSEA + MIMGO and “> (avg + std)” for all differentially expressed GO terms identified at GSEA *q*-value threshold values of 0.02 and 0.2. Click here for file

Additional file 2 **Table S2.** The Table shows all the true differentially expressed GO terms used in this study and the expression of their associated genes in the yeast cell cycle microarray data. Click here for file

## References

[B1] Woo Y Affourtit J Daigle S Viale A Johnson K Naggert J Churchill G A comparison of cDNA, oligonucleotide, and Affymetrix GeneChip gene expression microarray platforms J Biomol Tech 2004 15 276 284 15585824 PMC2291701

[B2] Edgar R Domrachev M Lash AE Gene Expression Omnibus: NCBI gene expression and hybridization array data repository Nucleic Acids Res 2002 30 207 210 10.1093/nar/30.1.207 11752295 PMC99122

[B3] Liang S Li Y Be X Howes S Liu W Detecting and profiling tissue-selective genes Physiol Genomics 2006 26 158 162 10.1152/physiolgenomics.00313.2005 16684803

[B4] Kadota K Ye J Nakai Y Terada T Shimizu K ROKU: a novel method for identification of tissue-specific genes BMC Bioinforma 2006 7 294 10.1186/1471-2105-7-294 PMC1501047 16764735

[B5] Ashburner M Ball CA Blake JA Botstein D Butler H Cherry JM Davis AP Dolinski K Dwight SS Eppig JT Harris MA Hill DP Issel-Tarver L Kasarskis A Lewis S Matese JC Richardson JE Ringwald M Rubin GM Sherlock G Gene ontology: tool for the unification of biology. The Gene Ontology Consortium Nat Genet 2000 25 25 29 10.1038/75556 10802651 PMC3037419

[B6] Kanehisa M Goto S KEGG: kyoto encyclopedia of genes and genomes Nucleic Acids Res 2000 28 27 30 10.1093/nar/28.1.27 10592173 PMC102409

[B7] da Huang W Sherman BT Lempicki RA Bioinformatics enrichment tools: paths toward the comprehensive functional analysis of large gene lists Nucleic Acids Res 2009 37 1 13 10.1093/nar/gkn923 19033363 PMC2615629

[B8] da Huang W Sherman BT Tan Q Kir J Liu D Bryant D Guo Y Stephens R Baseler MW Lane HC Lempicki RA DAVID Bioinformatics Resources: expanded annotation database and novel algorithms to better extract biology from large gene lists Nucleic Acids Res 2007 35 W169 W175 10.1093/nar/gkm415 17576678 PMC1933169

[B9] Subramanian A Tamayo P Mootha VK Mukherjee S Ebert BL Gillette MA Paulovich A Pomeroy SL Golub TR Lander ES Mesirov JP Gene set enrichment analysis: a knowledge-based approach for interpreting genome-wide expression profiles Proc Natl Acad Sci USA 2005 102 15545 15550 10.1073/pnas.0506580102 16199517 PMC1239896

[B10] Barry WT Nobel AB Wright FA Significance analysis of functional categories in gene expression studies: a structured permutation approach Bioinformatics 2005 21 1943 1949 10.1093/bioinformatics/bti260 15647293

[B11] Chen JJ Lee T Delongchamp RR Chen T Tsai CA Significance analysis of groups of genes in expression profiling studies Bioinformatics 2007 23 2104 2112 10.1093/bioinformatics/btm310 17553853

[B12] Mansmann U Meister R Testing differential gene expression in functional groups. Goeman’s global test versus an ANCOVA approach Methods Inf Med 2005 44 449 453 16113772

[B13] Dinu I Potter JD Mueller T Liu Q Adewale AJ Jhangri GS Einecke G Famulski KS Halloran P Yasui Y Improving gene set analysis of microarray data by SAM-GS BMC Bioinforma 2007 8 242 10.1186/1471-2105-8-242 PMC1931607 17612399

[B14] Efron BB Tibshirani R On testing the significance of sets of genes The Annals of Applied Statistics 2007 1 107 129 10.1214/07-AOAS101

[B15] Mar JC Matigian NA Quackenbush J Wells CA attract: A method for identifying core pathways that define cellular phenotypes PLoS One 2011 6 e25445 10.1371/journal.pone.0025445 22022396 PMC3194807

[B16] Yamada Y Hirotani K Satou K Muramoto K An identification method of data-specific GO terms from a microarray data set IEICE Trans Inf Syst 2009 E92-D 1093 1102 10.1587/transinf.E92.D.1093

[B17] Spellman PT Sherlock G Zhang MQ Iyer VR Anders K Eisen MB Brown PO Botstein D Futcher B Comprehensive identification of cell cycle-regulated genes of the yeast Saccharomyces cerevisiae by microarray hybridization Mol Biol Cell 1998 9 3273 3297 9843569 10.1091/mbc.9.12.3273 PMC25624

[B18] Gaddis ML Statistical methodology: IV. Analysis of variance, analysis of covariance, and multivariate analysis of variance Acad Emerg Med 1998 5 258 265 10.1111/j.1553-2712.1998.tb02624.x 9523936

[B19] Raponi M Belly RT Karp JE Lancet JE Atkins D Wang Y Microarray analysis reveals genetic pathways modulated by tipifarnib in acute myeloid leukemia BMC Cancer 2004 4 56 10.1186/1471-2407-4-56 15329151 PMC516036

[B20] Gatti DM Barry WT Nobel AB Rusyn I Wright FA Heading down the wrong pathway: on the influence of correlation within gene sets BMC Genomics 2010 11 574 10.1186/1471-2164-11-574 20955544 PMC3091509

